# The Natural History and Clinical Syndromes of Degenerative Cervical Spondylosis

**DOI:** 10.1155/2012/393642

**Published:** 2011-11-28

**Authors:** John C. Kelly, Patrick J. Groarke, Joseph S. Butler, Ashley R. Poynton, John M. O'Byrne

**Affiliations:** Department of Trauma and Orthopaedic Surgery, Cappagh National Orthopaedic Hospital, The Royal College of Surgeons in Ireland, Finglas, Dublin 11, Ireland

## Abstract

Cervical spondylosis is a broad term which describes the age related chronic disc degeneration, which can also affect the cervical vertebrae, the facet and other joints and their associated soft tissue supports. Evidence of spondylitic change is frequently found in many asymptomatic adults. Radiculopathy is a result of intervertebral foramina narrowing. Narrowing of the spinal canal can result in spinal cord compression, ultimately resulting in cervical spondylosis myelopathy. This review article examines the current literature in relation to the cervical spondylosis and describes the three clinical syndromes of axial neck pain, cervical radiculopathy and cervical myelopathy

## 1. Introduction

Cervical spondylosis is a broad term which describes the age-related chronic disc degeneration, which can also affect the cervical vertebrae, the facet, and other joints and their associated soft tissue supports. Chronic disc degeneration results in increased mechanical stressors passing through the cervical spinal column, resulting in osteophyte formation and secondary degenerative changes in surrounding structures, such as the facet joints, the posterior longitudinal ligament (PLL), and the ligamentum flavum. These degenerative changes and their associated nerve impingement are then responsible for the three clinical syndromes in which cervical spondylosis presents. Cervical spondylosis is generally classified according to these three clinical syndromes or means of presentation: axial neck pain, cervical radiculopathy, and cervical myelopathy. Patients can have a combination of any of the three syndromes. Evidence of spondylotic change is frequently found in many asymptomatic adults [1], with 25% of adults under the age of 40, 50% of adults over the age of 40, and 85% of adults over the age of 60 showing some evidence of disc degeneration [2, 3]. Another study of asymptomatic adults showed significant degenerative changes at 1 or more levels in 70% of women and 95% of men at age 65 and 60 [4]. The most common evidence of degeneration is found at C5-6 followed by C6-7 and C4-5 [3]. Treatment for mild and moderate disease is typically conservative with surgical intervention advised for those with severe intractable pain, progressive disease, and for those with associated weakness and neurological deficits.

## 2. Natural History

Cervical spondylosis can present itself in a multitude of ways. It can often be asymptomatic, it can can cause neck pain, regional pain, and can cause neurologic deficits of the sphincters, torso, or the extremities if there is spinal cord involvement [5].

In the majority of cases patients present between the ages of 40 and 60, with men being more commonly affected than women at a ratio of 3 : 2. 

Disc degeneration and bulging, osteophyte and spur formation, ligamentous hypertrophy, vertebral subluxation, decreased disc height, and facet joint arthropathy all combine to cause narrowing of the spinal canal and intervertebral foramina. Radiculopathy is a result of intervertebral foramina narrowing. Narrowing of the spinal canal can result in spinal cord compression, ultimately resulting in cervical spondylosis myelopathy. 

Factors which contribute to an acceleration in the disease process include having a congenitally narrow vertebral canal, certain athletic endeavors such as soccer, rugby, and horse riding, exposure to significant trauma, and having dystonic cerebral palsy which includes the cervical muscles [6–11]. 

The course of disease development and the ultimate prognosis for patients with cervical spondylosis is highly variable and extremely difficult to predict. In 1956 Clarke and Robinson longitudinally followed up 120 patients with cervical spondylosis myelopathy and a mean age of 53 years. They reported that in 75% of cases the disease deteriorated in an episodic manner, in 20% of cases there was a steady progression of symptoms, and in 5% of cases there was a rapid onset of symptoms followed by a long respite in disease progression [12]. 

In 1963 Lees and Turner longitudinally followed 44 patients with cervical spondylosis myelopathy from 3–40 years and again highlighted the disease's unpredictable nature. They found that “long periods of nonprogressive disability are the rule and a progressively deteriorating course is exceptional” and that “exacerbations can occur at long or shorter intervals for many years” [13].

Roberts reported on 24 patients and found that a long duration and severe symptoms were predictive of a poor outcome following operative intervention [14]. Nurick found in his study of 37 patients that there was an initial phase of deterioration followed by a longer nonprogressive phase which extended for many years, with older patients more likely to experience disease deterioration [8]. Epstein found that just over a third of patients improved, 38% remained stable, and 26% deteriorated [15]. Three studies from the same randomised controlled trials of conservative and operative treatment by Kadanka over 3 years, by Bedarnik over 2 years, and by Kadanka over 2 years found no difference between either group [16–18].

Based on these and other studies it seems that the progression of cervical spondylosis to cervical spondylosis myelopathy is highly variable and difficult to predict, with many patients experiencing a relatively benign form of the disease; however, a significant proportion of those presenting with neurological deficits do not experience spontaneous improvements and are subject to deterioration in their neurologic status over time [19].

## 3. Clinical Syndromes

### 3.1. Axial Neck Pain

Axial neck pain is the most common syndrome seen in clinical practice [20]. Improper posture, muscle fatigue, and poor ergonomics which occur as a consequence of muscular and ligamentous factors are major contributing factors to axial neck pain [21]. The aetiology of axial neck and shoulder pain is poorly understood and oftentimes there is no associated neurological deficit, making the disorder more difficult to treat. Many possible explanations have been given but their relative contribution has been difficult to quantify. In one study of patients with fibromyalgic neck pain, chemical analysis of the trapezius muscle from symptomatic adults found low levels of the high-energy phosphates, adenosine triphosphate, adenosine diphosphate and phosphoryl creatine, and an increase in the levels of adenosine monophosphate and creatine [22]. They concluded based on those results that muscle was the primary source of pain in those patients [22]. Larsson et al. demonstrated that patients with chronic trapezius myalgia had a lower muscle blood flow and higher intramuscular tension on their symptomatic side when compared to their asymptomatic side and when compared to asymptomatic controls [23]. Other factors such as a previous neck injury have been shown to be an independent and distinct risk factor for developing neck pain [24]. Facet joint arthropathy and cervical disc disease can also contribute to symptomatic neck pain. The synovium of facet joints and the peripheral portions of the intervertebral disc have been demonstrated to possess nerve fibres and nociceptive nerve endings [25–27]. Facet joint injection and discography have also assisted in providing evidence of their role in the aetiology of neck pain [28, 29]. One-third of patients with axial neck pain due to degenerative cervical spondylosis also present with headache, and greater than two-thirds present with shoulder pain which can either be unilateral or bilateral [30]. Many of these patients also present with arm, forearm, and hand pain. Another feature of this syndrome is chronic suboccipital pain which can radiate to the back of the ear, occiput, or neck and can be indicative of occipitoatlantal and atlantoaxial degeneration [30]. Restricted rotation of the head to one side can suggest involvement of the ipsilateral atlantoaxial joint [21].

### 3.2. Cervical Radiculopathy

The most commonly involved nerve roots in cervical radiculopathy are the sixth and seventh nerve roots which occur as a result of spondylosis of C5-C6 or C6-C7. Patients can present with arm pain, sensory deficits, neck pain, paraesthesia, reflex deficits, motor deficits, scapular pain, anterior chest pain, and, rarely, with left-sided chest and arm pain (cervical angina) [31, 32]. The symptoms of cervical radiculopathy are usually aggravated by performing the Spurling maneuver which describes extension or lateral rotation of the head to the side of the pain [21]. Davidson et al. demonstrated that relief from cervical monoradiculopathies due to extradural compressive disease may be obtained by performing the shoulder abduction sign, which involves elevating the arm overhead [33]. Radicular pain is thought to occur due to compression of an inflamed or irritated nerve root. In an animal model of chronic nerve root compression, Cornefjord et al. showed an increased concentration of the neurogenic chemical mediator of pain, substance P, in the dorsal root ganglia and the nerve root after 1 and 4 weeks [34]. Cooper et al. also demonstrated that chronic oedema and fibrosis within the nerve caused by compression can also increase the sensitivity of the nerve root to pain [35]. It has also been postulated that mechanical deformation of the dorsal root ganglia as occurs with a herniated disc causes a reduction in blood flow to the sensory nerve cell bodies resulting in pain [36]. Compression changes axonal flow which alters the metabolism of neurotransmitters within the axons and can then cause a decline in nerve function [37]. A prolapsed nucleus pulposus also initiates a local inflammatory response which results in the release of numerous inflammatory mediators such as tumour necrosis factor alpha (TNF-*α*), causing increased pain [38].

### 3.3. Cervical Myelopathy

Cervical myelopathy as a result of spondylosis is the most common cause of nontraumatic paraparesis and quadriparesis. It typically has an insidious onset and presents with clumsiness or reduced fine motor skills in the hands [20]. Patients complain of urinary urgency, hesitation, and frequency but rarely incontinence, and an increasingly awkward gait or difficulty maintaining balance, is frequently observed by family members [21]. Patients often present with neck stiffness and sometimes experience a stabbing pain in the preaxial or postaxial border of the arms [39]. Extension and flexion of the neck often elicits electric shock like sensations in the extremities with this known as Lhermitte's sign [20]. A more specific sign for CSM is Hoffman's sign. This sign is elicited by flipping either the volar or dorsal surfaces of the middle finger and observing the reflex contraction of the thumb and index finger. In particular, a dynamic Hoffman's sign may accentuate the reflex [40]. This is achieved by performing the test in different degrees of flexion and extension. Sung et al. reported that a positive Hoffman's sign strongly correlates with a cervical pathology [41].

Patients also demonstrate spasticity with exaggerated reflexes below the level of cord compression, motor weakness, sensory loss, and extensor plantar responses [20]. Some patients develop “myelopathy hand” which refers to a series of hand pathologies which include, loss of dexterity, diffuse numbness, intrinsic muscle wasting, ulnar and flexor drift of the ulnar two digits when trying to keep the fingers adducted and extended, and an inability to grasp and release the fist [21, 42, 43]. Mechanical compression of the spinal cord is thought to be the primary aetiological factor which results in myelopathy. In normal adults the anteroposterior diameter of the subaxial spine measures 17 to 18 mm. Those who have a diameter less than 13 mm are thought to have developmental stenosis and are predisposed to developing myelopathy [44]. 

There is a lot of evidence that suggests congenital narrow vertebral canals are related to developing cervical spondylosis myelopathy (CSM) [45]. The Torg-Pavlov ratio is a relativity straightforward measurement that is taken from lateral radiographs of the cervical spine. It is calculated by dividing the width of the spinal canal at a level (taken from the midpoint of posterior surface of the vertebral body to the closest point in the junction of the lamina and spinous process), by the diameter of the vertebral body at that level. Yue et al. in a study measuring the Torg-Pavlov ratio of 1130 individuals, showed that the ratio is significantly lower in patients with cervical spondylotic myelopathy compared with a nonspondylotic, nonmyelopathic control group, irrespective of sex and age [46].

Other factors which play a part in myelopathy development include a cross-sectional area less than 60 mm^2^ and a banana-shaped cord [47, 48]. Another important factor in the development of significant neurologic deficit is having an anteroposterior cord compression ration of less than 40% and this is suggestive of significant flattening of the cord and a worse prognosis [43]. Normal neck movements can change the dimensions of the spinal canal and can assist in myelopathy development by causing cord compression [21]. The volume of the cervical spinal canal and the anteroposterior diameter have been shown to be reduced in extension [49, 50]. The spinal cord has also been shown to stretch with flexion of the cervical spine and shorten and thicken with extension [51]. This thickening in extension then exposes the cord to increase compressive forces from the lamina or the ligamentum flavum. 

Instability is another important consideration in patients with CSM. Some authors consider severe disc degeneration as being equivalent to “autofusion” as a compensation process for segmental instability. Wang et al. recommend multilevel anterior cervical decompression and fusion (ACDF) or expansive laminoplasty as a surgical management of CSM with severe disc degeneration because of the associated instability [52].

Cervical spondylotic myelopathy has also been shown to affect patients' quality of life with over one-third of patients having increased anxiety or depression as a result of their reduced mobility [53].
